# Transient rRNA synthesis inhibition with CX-5461 is sufficient to elicit growth arrest and cell death in acute lymphoblastic leukemia cells

**DOI:** 10.18632/oncotarget.5413

**Published:** 2015-10-12

**Authors:** Sandeep S. Negi, Patrick Brown

**Affiliations:** ^1^ Department of Oncology and Pediatrics, Johns Hopkins University School of Medicine, Baltimore, MD 21231, USA

**Keywords:** CX-5461, rRNA synthesis, UCN-01, acute lymphoblastic leukemia, MAP kinase

## Abstract

Enhanced rRNA synthesis is a downstream effect of many of the signaling pathways that are aberrantly activated in cancer, such as the PI3K/mTOR and MAP kinase pathways. Recently, two new rRNA synthesis inhibitors have demonstrated therapeutic effects on cancer cells while sparing normal cells. One of them, CX-5461, is currently in phase 1 clinical trials for hematological malignancies. Here, we investigate the effectiveness of transient treatment with this drug on acute lymphoblastic leukemia cells. Our results show that short exposure to CX-5461 followed by drug washout is sufficient to induce persistent G2 cell-cycle arrest and irreversible commitment to cell death, in spite of rRNA synthesis returning to normal within 24 hours of drug washout. The magnitude of cell death after transient exposure is similar to continuous exposure, but the time to cell death is relatively delayed with transient exposure. In this report, we also investigate rational drug combinations that can potentiate the effect of continuous CX-5461 treatment. We show that the checkpoint abrogator UCN-01 can relieve CX-5461-induced G2 arrest and potentiate the cytotoxic effects of CX-5461. Finally, we show that ERK1/2 is activated upon CX-5461 treatment, and that pharmacological inhibition of MEK1/2 leads to enhanced cell death in combination with CX-5461. In summary, our results provide evidence for the effectiveness of CX-5461 pulse treatment, which may minimize drug related toxicity, and evidence for enhanced effectiveness of CX-5461 in combination with other targeted agents.

## INTRODUCTION

The primary role of the nucleolus is to synthesize ribosomes. However, identification of the nucleolar proteome showed that a majority of nucleolar proteins have functions unrelated to ribosome biogenesis, such as cell-cycle regulation and stress response, [1, 2] and subsequent studies have discovered novel functions for the nucleolus both in normal and disease states [3]. The nucleolus is a dynamic structure which is formed by the act of making ribosome. Nucleolar stress can be caused by inhibition of any of the many steps involved in ribosome biogenesis including pre-ribosomal RNA (rRNA) synthesis, rRNA processing, and assembly of ribosomal proteins on nascent rRNA transcripts and export of mature ribosome from the nucleus to the cytoplasm [4]. Nucleolar stress caused by drugs or environmental factors can lead to unraveling of the nucleolus and the release of nucleolar proteins into the nucleoplasm. Rubbi *et al.* [5] first proposed that impairment of nucleolar function in response to cellular stress leads to p53 activation, which in turn leads to cell-cycle arrest or apoptosis.

Ribosome biogenesis is a highly coordinated process that is regulated by tumor suppressor proteins and oncogenes [6]. Morphological and structural changes in the nucleolus were one of the earliest reported markers in cancer. RNA polymerase I (RNA pol I) is responsible for the synthesis of pre-rRNA. Elevated RNA pol I activity due to increased growth and protein synthesis demand is a hallmark of cancer [6, 7]. In fact, some of the major signaling pathways deregulated in cancers directly affect ribosome biogenesis. Among them, c-Myc and PI3K-AKT-mTOR signaling directly regulate multiple steps in ribosome biogenesis [8, 9]. As ribosome biogenesis is an essential cellular process for normal cells, its therapeutic targeting in cancer seems unlikely. However, recently, a class of drugs targeting rDNA transcription has shown promise as novel cancer treatment in pre-clinical models [10, 11, 12, 13, 14, 15]. These studies have shown that therapeutically inhibiting rDNA transcription with these drugs selectively kills cancer cells and spares normal cells.

CX-5461 is the first potent and selective inhibitor of RNA pol I transcription [16]. Recently, the rRNA synthesis inhibitors, CX-5461 and BMH-21, have shown therapeutic potential in different cancer models [10, 13, 17]. These drugs have distinct mechanisms of action of inhibiting rRNA synthesis. BMH-21 was initially discovered as an activator of p53, and was later found to induce nucleolar stress by inhibiting RNA pol I binding to the rDNA promoter and decreased rRNA synthesis [13, 18]. In contrast, CX-5461 inhibits the interaction between SL1 and rDNA thereby preventing the formation of pre-initiation complex. Bywater *et al.* [10] showed therapeutic potential of CX-5461 treatment in mouse model of melanoma and MLL-AF9 acute myeloid leukemia. Their work showed that nucleolar stress caused by CX-5461 selectively led to p53 activation and subsequent apoptosis in cancer cells. Recently, we have shown that CX-5461 arrests acute lymphoblastic leukemia (ALL) cells in G2 phase and induces apoptosis in p53 independent manner [19].

In recent years, potent but transient inhibition of BCR-ABL kinase in CML, and PI3K in breast cancer models has been shown to be an effective therapeutic strategy [20, 21, 22]. Here, we investigated the cellular response to transient inhibition of rRNA synthesis with CX-5461 treatment. We found that short exposure to CX-5461 produces similar effects as seen with continuous treatment. Despite reactivation of rRNA synthesis activity within 24 h of drug washout, transient and potent inhibition of rRNA synthesis with CX-5461 was sufficient to commit ALL cells to irreversible cell death. Apart from acute treatment strategy, we also investigated rational drug combinations that can enhance the cytotoxicity of continuous CX-5461 treatment. In this report we analyzed the effect of inhibiting cellular pathways activated by CX-5461 treatment. We showed that checkpoint kinase inhibitor UCN-01 and MAPK pathway inhibitors enhance CX-5461 mediated cytotoxicity.

## RESULTS

### Transient exposure to CX-5461 is cytotoxic

**Figure 1:**
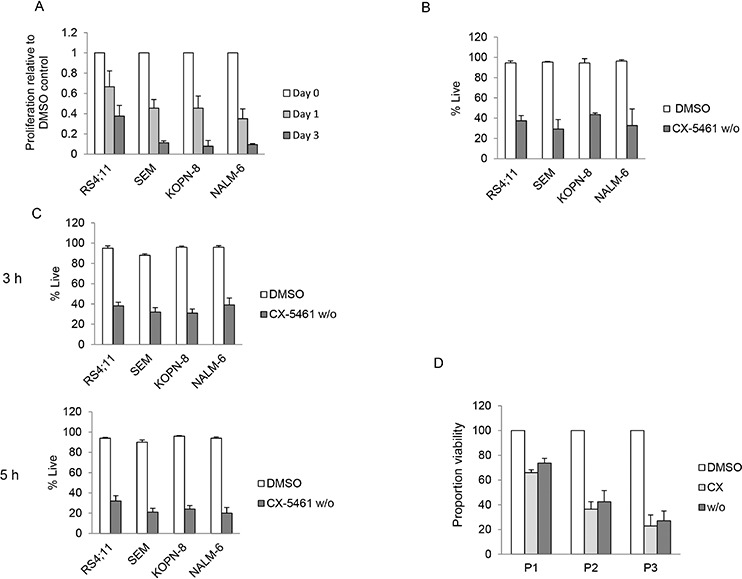
Transient inhibition of rRNA synthesis affects cell proliferation **A.** Four ALL cell lines were treated with 250 nM CX-5461 or DMSO for 24 h. Cells were washed and equal number of CX-5461 or DMSO treated cells were seeded in drug free medium in 96 well plates and cell proliferation was measured at Day 1 and 3. Data is normalized to the growth in DMSO treated samples. All four ALL cell lines show time dependent decrease in proliferation relative to their DMSO treated controls. Data represents mean +/− S.D. of three independent experiments. **B.** Cells were treated as in (a) and cell death was measured three days after washout by propidium iodide staining (PI). Data represent mean +/− S.D. of three independent experiments. **C.** Cells were treated for 3 hours or 5 hours with CX-5461 (500 nM for NALM-6 and 250 nM for SEM, KOPN-8 and RS4;11) or DMSO followed by washing. Cells were incubated in drug free media and cell viability was measured using trypan blue after three days. Drug washout cells show reduced viability compared to control treated cells. Data represent mean +/− S.D. of three independent experiments. **D.** Three ALL patient samples were treated with 1 μM CX-5461 or DMSO for 5 hours. After 5 hours the CX-5461 treated cells were washed, incubated with either DMSO (w/o) or 1 μM CX-5461 (CX); the DMSO treated cells were washed and incubated in DMSO (DMSO). After 2 days, cell death was measured using PI staining. All three patient samples, continuously or transiently treated with CX-5461, showed reduced viability compared to DMSO treated control. Viable proportion is plotted from duplicate experiments.

We first established a washout procedure to evaluate whether transient exposure to CX-5461 is sufficient to irrevocably induce cell death in ALL cells. Cells were treated with 250 nM CX-5461 or DMSO for 24 hours, washed twice in the culture medium and suspended in drug free medium. We measured cell proliferation using the colorimetric MTS assay at day 1 and 3 after resuspension. All cell lines showed a time dependent reduction in cell proliferation in washout cells relative to control treated cells (Figure 1A). To assess the extent to which reduced proliferation was due to induction of cell death (as opposed to growth arrest only), we measured cell death at day 3 after washout using FACS after staining with propidium iodide (PI). All cell lines showed significant reduction in proportion of live cells (i.e., PI negative) in washout cells compared to DMSO treated controls after 3 days (Figure 1B). To investigate if a shorter incubation with CX-5461 would still result in cytotoxicity, we exposed the cells to CX-5461 for 3 hours and 5 hours. We measured cell viability using trypan blue 4 days after washout. All cell lines showed a reduction in viability in drug washout cells (Figure 1C). We then investigated if transient exposure would lead to cytotoxicity in primary patient samples. We have previously shown that normal bone marrow cells show minimal cell death when treated with 1 μM CX-5461 for two days [19]. For transient exposure, we treated patient samples (*n* = 3) for 5 hours with 1 μM CX-5461, washed them twice and resuspended in drug free media. Cell death was measured with PI staining. All three samples showed reduced viability in drug washout, and to a similar extent as with continuous treatment compared to DMSO treated controls (Figure 1D). Taken together, these results show that short exposure to CX-5461 is sufficient to induce cell death in acute leukemia cells.

### rRNA synthesis recovers in drug washout cells

**Figure 2:**
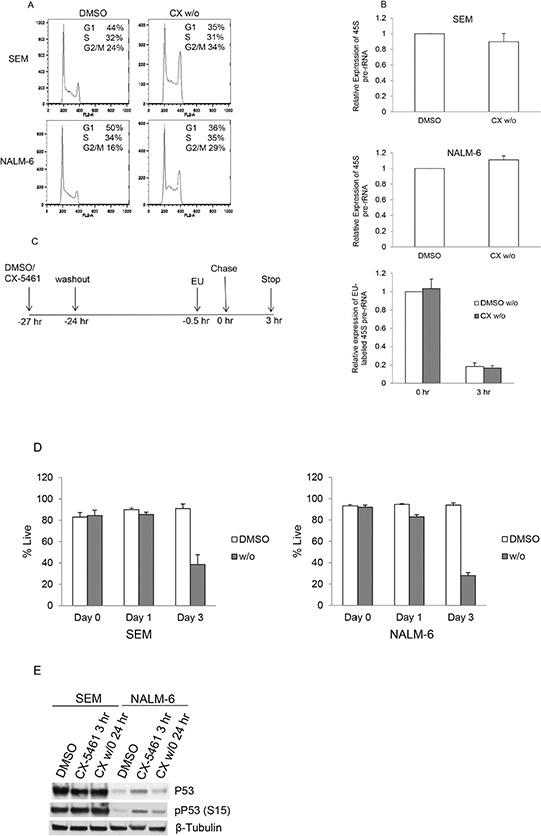
Transient potent rRNA synthesis inhibition with CX-5461 is sufficient to commit ALL cells to cell death despite reactivation of rRNA synthesis **A.** SEM and NALM-6 cells were treated with 250 or 500 nM CX-5461, respectively. An aliquot was harvested after 3 hours, washed twice and cells were suspended in drug free media. Cell-cycle distribution was analyzed after 24 hours by flow cytometry of PI stained cells. Cells show aberrant cell-cycle distribution in drug washout cells compared to DMSO treated control cells. Representative flow cytometry data is shown from one of the three experiments. **B.** 45S pre-rRNA transcript levels were measured using quantitative PCR and normalized to the expression of GAPDH and Actin. DMSO and CX-5461 washout cells (CX w/o) show no difference in pre-rRNA synthesis at 24 hours. Experiments were repeated three times and data represents mean +/− S.D. **C.** Schematic of EU labeling of drug washout SEM cells. Newly synthesized EU labeled 45S pre-rRNA transcript levels were measured at 0 and 3 hours after EU removal. **D.** Cells were treated as in (a) and cell viability was measured using trypan blue staining. Drug washout cells show reduced viability compared to DMSO treated cells. Experiments are repeated three times. Data represents mean +/− S.D. **E.** SEM and NALM-6 cells were treated as before. NALM-6 cells show an increase in p53 and phospho-p53 levels at 3 hours after CX-5461 treatment. Elevated p53 levels in NALM-6 cells were substantially reduced 24 hours after drug washout.

To further investigate changes induced by transient treatment, we treated SEM and NALM-6 cells with CX-5461 for 3 hours, washed twice and resuspended them in drug free media. We then investigated the effects of drug washout on cell-cycle distribution, rRNA synthesis and cell viability. Cell-cycle results show that 24 hours after washout (CX w/o), cells show an increase in the G2/M population compared to control treated cells, although the magnitude of the increase is less than that seen with continuously treated cells (CX-5461) (Figure 2A and Supplementary Figure 1A). We used 45S pre-rRNA transcript levels, which are known to have a very short half-life (several minutes), as a measure of the rate of rRNA synthesis. We have shown previously that 250 and 500 nM CX-5461 reduces pre-rRNA synthesis by more than 50% by 3 hours in SEM and NALM-6 cells respectively [19]. We first measured 45S pre-rRNA levels at 3 hours after CX-5461 treatment to confirm inhibition of RNA pol I transcription (Supplementary Figure 1B). The cells were then washed and suspended in drug free media for 24 hours to check if pre-rRNA synthesis recovered following washout. Both cell lines showed significant pre-rRNA synthesis inhibition at 3 hours (CX 3 h) and almost complete recovery at 24 hours after washout (CX w/o) (Figure 2B and Supplementary Figure 1B). Ribosome biogenesis is a highly coordinated process and inhibition of rRNA synthesis can lead to pre-rRNA processing defects. In order to make sure that the increase in the levels of 45S pre-rRNA in drug washout cells is not due to pre-rRNA processing defects, we labeled SEM cells with ethynyl uridine (EU) for 30 min followed by chase in EU free media. RNA was isolated at 0 and 3 hours after EU washout from the cells. Newly synthesized EU labeled transcripts were isolated as described in materials and methods. Our results show no difference in the levels of newly transcribed 45S pre-rRNA in DMSO and CX-5461 washout cells at 0 hour (Figure 2C). Moreover, 3 hours after chase, levels of the EU labeled 45S pre-rRNA decreases substantially. The decrease was similar in both DMSO and CX-5461 washout cells suggesting efficient processing of 45S pre-rRNA transcript under both conditions.

Next, we measured cell viability of these cells after washout at day 1 and 3 using trypan blue. The results show that transient inhibition of rRNA synthesis substantially decreased cell viability (Figure 2D). These results confirm that despite reactivation of rRNA synthesis activity within 24 hours of drug washout, short-term rRNA synthesis inhibition with CX-5461 was sufficient to inhibit cell cycling and viability. We have previously shown that p53 levels were increased upon 24 hours CX-5461 treatment in p53 wild-type cell lines, although cell-cycle arrest and apoptotic effects were p53-independent [19]. To investigate if p53 levels remains elevated in CX-5461 washout cells, we measured p53 and phospho-p53 levels in drug treated and drug washout cells (Figure 2E). As shown previously [19], there was no increase in p53 levels in SEM cells (p53 mutant cell line). In NALM-6 cells (p53 wild-type cell line), there was an increase in p53 and phospho-p53 levels initially at 3 hours after drug treatment but drug washout resulted in a decrease in p53 levels when measured after 24 hours.

### Washout effectively removes residual drug from the culture medium

**Figure 3:**
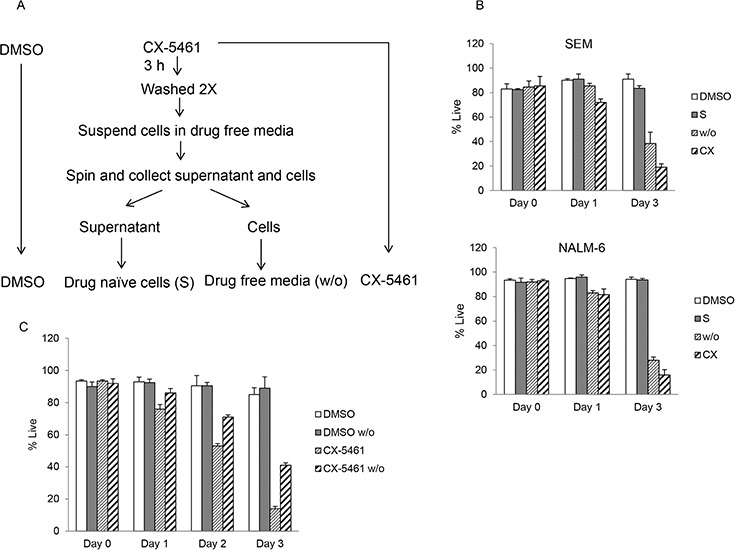
Washout procedure completely removes drug from the media **A.** Schematic of drug treatment experiment in (b). SEM and NALM-6 cells were continuously treated with DMSO or with 250 or 500 nM CX-5461 (CX) respectively. A portion of CX-5461 treated cells was harvested after 3 hours, washed twice and incubated in drug free media. After second wash, cells were suspended in drug free medium and spun again. Resulting supernatant was collected and added to drug naïve cells (S) while the cells pellet was suspended in fresh media (w/o). **B.** Cell viability was measured immediately after washout and at day 1 and 3 with trypan blue staining. Experiment was performed three times and mean +/− S.D. is plotted. **C.** SEM cells were continuously incubated in DMSO or CX-5461 and a portion was harvested after 3 hours followed by washing in drug free media as before. Cell viability was measured using trypan blue staining at day 0, 1, 2 and 3 after washout. Results are plotted as mean +/− S.D.

We considered the possibility that the cytotoxic effect seen after drug washout is due to incomplete removal of the drug. To test for any residual drug, the cell culture supernatant (S) from drug treated cells after two washes was transferred to previously untreated cells (naïve) (Figure 3A). These cells were allowed to grow for three days and cell viability was measured by trypan blue staining. Our results show no reduction in viability of cells incubated in media from drug wash out cells similar to untreated control cells (Figure 3B). These results show effective removal of the drug from the culture media following two washes. We further tested the effect of washout itself on cell viability. SEM cells were treated with CX-5461 or DMSO for 3 hours followed by two washes in drug free media. As shown in Figure 3C, washout has no effect on cell viability as DMSO washout cell show similar viability as control unwashed cells. Also, these results suggest a lag in cell death in drug washout cells compared to continuously treated cells (Figure 3C).

### UCN-01 treatment relieves cell-cycle arrest and shows enhanced cell killing in combination with CX-5461

**Figure 4:**
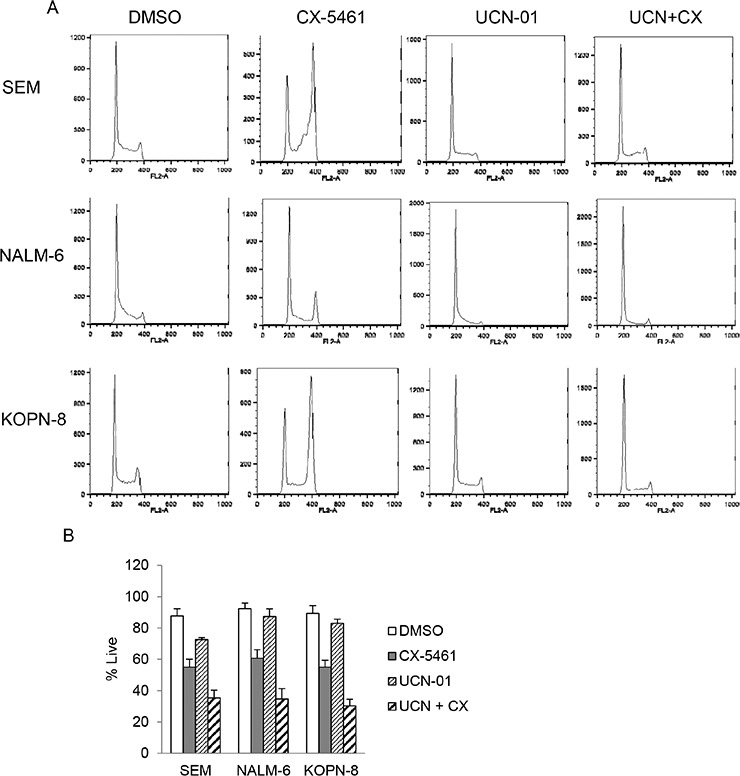
UCN-01 relieves CX-5461 induced G2/M phase arrest **A.** Cells were either treated with CX-5461 and UCN-01 alone or pretreated with 100 nM UCN-01 for 1 hour followed by 250 nM CX-5461 for 1 day. Cell-cycle distribution was determined by flow cytometry analysis of PI stained cells. UCN-01 completely removed G2/M block induced by CX-5461. One representative experiment out of three is shown. **B.** Cells were treated as in (a) and cell viability was measured at 55 hours post drug treatment using trypan blue staining. Combination treatment shows enhanced cytotoxicity compared to treatment with single agent. Experiment was repeated three times and mean +/− S.D. is plotted.

In addition to transient treatment with CX-5461, we investigated other rational drug combinations that can potentiate the effect of continuous CX-5461 treatment. We have previously shown that CX-5461 activates ATM/ATR pathway in acute leukemia, arrests cells in G2 phase and synergizes with ATR inhibitor in killing these cells [19]. We hypothesized that abolishing cell-cycle arrest in the G2 phase would result in inadequate recovery from cellular stress and lead to increased cell death. Checkpoint kinases 1 and 2 (CHK1/2) are downstream effectors of ATM/ATR pathway and are activated upon CX-5461 treatment [19]. UCN-01, a staurosporine analogue, is a potent inhibitor of CHK1/2 and sensitizes tumor cells to genotoxic agents by abrogating G2 arrest induced by these drugs [23, 24]. Although UCN-01 is an inhibitor of the protein kinase C (PKC) family of enzymes, G2 checkpoint abrogation by inhibiting checkpoint kinases play a major role in its anticancer activity [25, 26, 27]. To determine if UCN-01 can abrogate CX-5461 induced G2 arrest, we pre-treated cells with UCN-01 followed by continuous CX-5461 treatment for 24 hours. Cell-cycle analysis shows that CX-5461 treatment induced G2 arrest in SEM, KOPN-8 and NALM-6 cells. This arrest was completely abolished by UCN-01 pre-treatment (Figure 4A). We then investigated if UCN-01 treatment will enhance the cytotoxicity of CX-5461 as seen previously in combination with ATR inhibitor. We pre-treated cells with UCN-01 for 1 hour followed by CX-5461 and measured cell viability using trypan blue. Combination treatment with CX-5461 and UCN-01 significantly reduced cell viability in all three cell lines compared to CX-5461 or UCN-01 treatment alone (Figure 4B).

### CX-5461 activates MAPK signaling pathway

**Figure 5:**
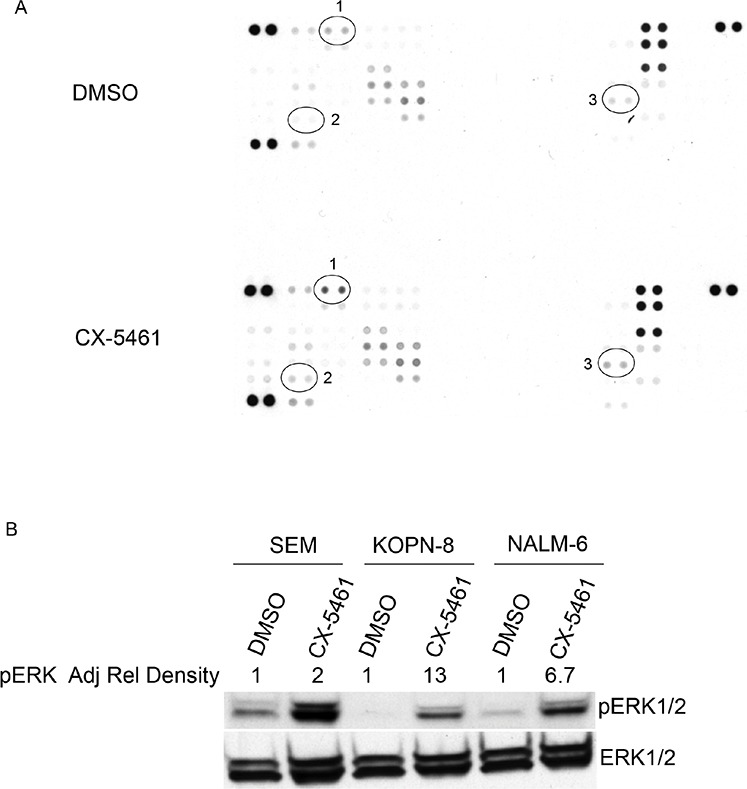
CX-5461 activates MAPK signaling pathway **A.** SEM cells were treated with 250 nM CX-5461 or DMSO for 1 day. Proteome profiler human phospho-kinase array was incubated with equal amount of control or drug treated sample. Results show an increase in pERK (1), pCHK2 (2) and HSP60 (3) signal in CX-5461 treated cells compared to DMSO treated control. **B.** Increase in pERK signal was confirmed with western blot of CX-5461 treated SEM, NALM-6 and KOPN-8 cells. Adjusted relative density of pERK signal normalized to corresponding DMSO treated control is indicated.

**Figure 6:**
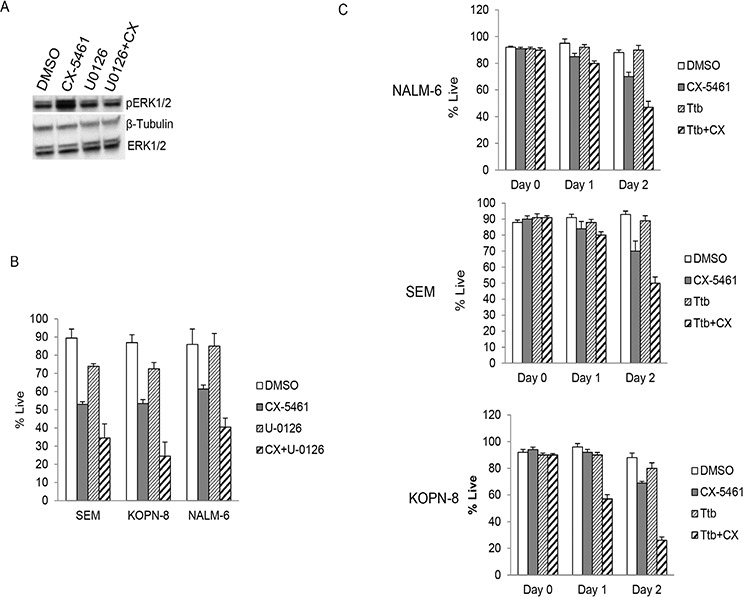
MEK1/2 inhibitors enhance cytotoxicity of CX-5461 **A.** SEM cells were treated with 250 nM CX-5461 alone or 10 μM U-0126 alone or their combination. Western blot shows U-0126 reduced the levels of pERK induced by CX-5461 treatment. **B.** SEM, KOPN-8 and NALM-6 cells were treated as in (a) and cell viability was measured using trypan blue staining at 55 hours. **C.** Cell lines were treated as in (a) but with another MEK1/2 inhibitor trametinib (150 nM Ttb). Combination treatment showed reduced viability in all three cell lines compared to single agent treated cells. (b, c) All experiments were repeated three times. Data represents mean +/− S.D.

As ribosome biogenesis is a highly regulated process, we investigated other signaling pathways activated by rRNA synthesis inhibition. We incubated a human phospho-kinase protein array with equal amounts of cell lysates from SEM cells treated with CX-5461 or DMSO for one day. CX-5461 treated cells showed an increase in pERK1/2 (Thr202/Tyr204), pCHK2 (T68) and HSP60 levels (Figure 5A). We further confirmed the activation of ERK1/2 with western blot from CX-5461 treated SEM, NALM-6 and KOPN-8 cells (Figure 5B). We investigated if pharmacological inhibition of the MEK/ERK signaling pathway would abrogate CX-5461 induced ERK1/2 activation and potentiate the effect of rRNA synthesis inhibition. We treated SEM cells with MEK1/2 inhibitor, U-0126, in combination with CX-5461 for one day. Western blot results show that U-0126 reduced the levels of pERK induced by CX-5461 treatment (Figure 6A). More-over, cell viability was substantially reduced in cells treated with a combination of U-0126 and CX-5461 compared to CX-5461 or U-0126 alone (Figure 6B). To further confirm that treatment with MEK/ERK inhibitor can enhance CX-5461 cytotoxic effect, we treated ALL cell lines with another MEK1/2 inhibitor, trametinib. Cells were treated with 150 nM trametinib in combination with CX-5461 for 2 days and cell viability was measured by trypan blue staining. As seen with U-0126, trametinib treated cells show significant increase in cell death in combination with CX-5461 then cells treated with single agent (Figure 6C).

## DISCUSSION

In cancer therapy, continuous target inhibition has been seen as a pre-requisite for maximum clinical impact. Our results show that transient treatment with CX-5461 induces cellular changes similar to continuous treatment, albeit with a lag period. Importantly, after drug washout, cells are irreversibly committed to cell death despite complete recovery from rRNA synthesis inhibition. This suggests that short term blockade of rRNA synthesis is sufficient to irreversibly inhibit cellular proliferation. The rationale for continuous target inhibition for maximum efficacy has been challenged in chronic myeloid leukemia (CML). A series of studies have shown that cytotoxicity in CML cells can be achieved with transient potent BCR-ABL inhibition [20, 21]. Dasatinib, a second-generation BCR-ABL kinase inhibitor, with a short half-life of approximately 3–5 hours has been shown to be clinically effective with once-daily administration despite only intermittent BCR-ABL inhibition [28]. More-over, tolerability of once-daily dosing was superior to the twice-daily schedule even with partial kinase inhibition. Recently, transient inhibition of PI3K in breast cancer cells was shown to be an effective therapeutic strategy [22].

Yung *et al.* [29] have reported that low dose actinomycin D treatment for short duration followed by washout leads to complete recovery of cell growth and rRNA synthesis, whereas higher dose or longer duration lead to irreversible inhibition of rRNA synthesis and cell proliferation. Recently, Ma *et al.* [30] showed similar results on cell-cycle arrest with actinomycin D treatment as seen with CX-5461. But in contrast to our results, they showed more than 80% inhibition of rRNA synthesis at 20 hours post washout after a 2 and 4 hour actinomycin D treatment. Compared to their results, we achieved 50% inhibition in 3 hours with CX-5461. It is possible that higher inhibition may lead to irreversible repression of rRNA synthesis. Another explanation may be the use of a solid cancer cell line in their study. This difference may also be due to a different mechanism of action. Actinomycin D is a RNA polymerase inhibitor which intercalates into GC rich regions of rDNA and shows selectivity for RNA pol I at low dose. It inhibits Pol I transcription during the elongation step whereas CX-5461 disrupts the binding of the SL1 transcription factor to rDNA promoter, which inhibits initiation of rRNA synthesis by the Pol I complex. Nevertheless, in our case, recovery from rRNA synthesis after washout did not change the eventual fate of these cells.

One of the most studied effects of nucleolar stress is the stabilization of p53 resulting in cell-cycle arrest and/or apoptosis [5]. ARF tumor suppressor has been shown to translocate to the nucleoplasm in response to nucleolar stress where it inhibits the binding of E3 ubiquitin ligase, MDM2, to p53 resulting in p53 stabilization [31]. Recent reports have shown that drugs targeting rRNA synthesis activate a p53-dependent apoptosis pathway in cancer cells displaying high rate of ribosome biogenesis [10, 32]. Although p53 activation upon ribosomal stress is well established, there are reports that provide evidence for a p53-independent mechanism that links nucleolar stress to inhibition of cell proliferation. We have previously shown that rRNA synthesis inhibition by CX-5461 activates ATM/ATR kinase pathway leading to CDC2 phosphorylation, G2 arrest and apoptosis in both p53 mutant and wild-type acute leukemia cells [19]. In line with that report, here we showed that p53 is activated upon 3 hours treatment in p53 wild-type cell line but the levels go down within 24 hours after drug washout suggesting p53-independent downstream effects of CX-5461. Donati *et al.* [33] showed that knockdown of POLR1A gene, which encodes the catalytic subunit of RNA polymerase I, in p53 null cells leads to cell-cycle arrest due to the down-regulation of transcription factor E2F-1. Ribosomal stress can also reduce the levels of PIM1 kinase leading to inhibition of cell proliferation in p53 null cells by stabilizing cell-cycle inhibitor p27^kip1^, a target of PIM1 kinase [34]. This reduction in PIM1 levels can be seen as early as 3 hours after rRNA synthesis inhibition, a time frame similar to one used in this study.

Many proteins involved in stress response, proliferation and cell-cycle progression are sequestered in the nucleolus (away from their site of action or interacting partners) thereby controlling their action [31]. At the onset of mitosis, rRNA synthesis is suppressed and nucleolus is disassembled in a highly regulated fashion. Many of the nucleolar proteins are phosphorylated by CDC2/Cyclin B complex (including members of rRNA synthesis and processing machinery) and are dissociated from the nucleolus [35]. One interesting question then is why transient inhibition of rRNA synthesis by CX-5461 affects cellular proliferation but suppression of rRNA synthesis during mitosis does not. We speculate that the untimely release of proteins sequestered in the nucleolus, upon drug treatment, results in cell-cycle arrest and apoptosis. For example, tumor suppressor protein ARF is sequestered in the nucleolus in association with NPM1 [36]. On nucleolar disruption by drug treatment or radiation, ARF translocates to the nucleoplasm, binds to E3 ligase MDM2 thereby preventing p53 ubiquitination. Elevated p53 levels then lead to cell-cycle arrest or apoptosis depending on the level of cellular insult [31]. Interestingly, ARF levels decrease during mitosis and recover in early G1 phase [37]. ARF has also been shown to inhibit growth in p53-independent manner by arresting cells in G2 phase which subsequently leads to apoptosis [38]. Also, we cannot rule out the possibility that CX-5461 has other targets in the cells which stay inhibited even after drug removal.

We have previously shown that caffeine and an ATR inhibitor can relieve CX-5461 induced G2 arrest, eventually leading to enhanced apoptosis [19]. Here, we showed that CX-5461 induced G2 arrest can be abolished by the checkpoint inhibitor UCN-01 [23, 26, 39] which also leads to enhanced cell death. This suggests that relieving G2 arrest by checkpoint kinase inhibitor UCN-01 provides no opportunity to the cells to overcome stress induced by CX-5461 treatment. As UCN-01 has been shown to enhance the cytotoxicity of radiation and chemotherapy, combination treatment with UCN-01 represents a therapeutic strategy that can potentiate the effectiveness of CX-5461 [40, 41]. More-over, CX-5461 treatment activates MAP kinase pathway and MEK inhibitors showed increased cell killing in combination with this rRNA synthesis inhibitor (Supplementary Figure 1C). In summary, our data suggests that transient inhibition of rRNA synthesis is sufficient to activate irreversible changes in cell survival and supports the potential for pulse treatment strategy in treating ALL with CX-5461, which in turn may minimize drug related toxicity. Also, we have provided *in vitro* evidence that rational combinations of CX-5461 with other inhibitors of survival pathways activated upon inhibition of rRNA synthesis can potentiate its effectiveness and should be further investigated in an *in vivo* model system.

## MATERIALS AND METHODS

### Cell lines and patient samples

RS4;11, SEM, KOPN-8 and NALM-6 cell lines were purchased from German Collection of Microorganisms and Cell Cultures (DSMZ). Informed consent was obtained from patients, in accordance with the institutional review board guidelines, for the samples used in this study. Blasts were isolated from patient samples using Ficoll-Hypaque density gradient centrifugation and stored in liquid nitrogen for future use. The diagnosis of ALL was based on morphology and flow cytometry data. Cytogenetic was determined by standard procedures. Cell lines and patient samples used in this study are

**Table:** 

Patient Sample	Cyto-genotypes	
P1	MLL-AF4	
P2	TEL-AML	
P3	MLL-ENL	
Cell Line	Cyto-genotypes	p53 status
RS4;11	MLL-AF4	wild-type
SEM	MLL-AF4	mutant
KOPN-8	MLL-ENL	mutant
NALM-6	t(5;12)(q33.2;p13.2)	wild-type

### Drug treatment and washout

Cells were incubated with CX-5461 for indicated time. Cells were washed twice in culture media and reseeded in drug free media. For experiments with drug naïve cells, CX-5461 treated cells were washed twice and suspended in drug free media. The cells were centrifuged again, supernatant were collected and this supernatant was added to drug naïve cells (denoted as “S” in Figure 3B). Cell viability was measured using trypan blue staining or flow cytometry of PI stained cells. CX-5461 was purchased from Xcess Biosciences; UCN-01 and U-0126 from Sigma-Aldrich; Trametinib from LC laboratories.

### Cell proliferation

Cells were treated with DMSO or CX-5461 for 24 hours, washed twice and equal numbers of cells were seeded in 96 well plates. Cell proliferation was measured at 0 hour, 24 hours and 72 hours after washout using CellTiter 96 AQueous One Solution Cell Proliferation solution (MTS reagent) (Promega). MTS reagent was added to each well and incubated for 1 hour at 37°C in dark and absorbance was recorded at 490 nm using Bio-Rad microplate reader. Results were background subtracted and normalized to DMSO treated control.

### Flow cytometry

Cells were fixed in methanol and stored at −20°C until further processing. For cell-cycle analysis cells were spun down, washed in PBS and incubated in RNaseA containing propidium iodide (PI) solution at 37°C for 30 min in dark. Cells were run on BD FACScaliber (BD Biosciences) and cell-cycle analysis was performed using FlowJo software (Tree Star).

### Phospho kinase proteome array and western blotting

Phospho kinase levels were measured using Proteome Profiler Human Phospho-Kinase Array kit as suggested by the manufacturer (R&D System). Briefly, cells were lysed and protein concentrations were measured. Each phospho kinase array was incubated with 200 μg of protein lysate from DMSO or CX-5461 treated cells. Array was developed according to manufacturer's instructions. For western blots, cell lysates were run on SDS Polyacrylamide gel and transferred to PVDF membrane. Membrane was blocked with 5% milk and incubated with primary antibody against ERK, Phospho-p44/42 MAPK (Erk1/2) (Thr202/Tyr204) and β-Tubulin. Antibodies were purchased from Cell Signaling Technology.

### EU labeling of RNA transcript

Pre-rRNA synthesis and processing of 45S transcript was determined by Click-iT^®^ Nascent RNA Capture Kit (Life Technologies) according to manufacturer's instructions [42]. Briefly, 0.4 mM ethynyl uridine (EU) was incorporated into SEM cells for 30 min. Cells were allowed to recover in EU free media and RNA was isolated at 0 and 3 hours after washout. EU-labeled RNA was biotinylated and captured on streptavidin magnetic beads according to the protocol. cDNA synthesis was performed directly on the beads. Relative levels of 45S pre-rRNA were measured by qPCR at each time point. All experiments were performed in triplicates.

### qPCR

Cells were treated with indicated concentrations of CX-5461. A portion of cells were harvested after 3 hours drug exposure and RNA was isolated. Rest of the cells were washed twice and reseeded in drug-free culture media. RNA was isolated, using RNeasy mini kit (Qiagen), from these cells after 24 hours. qPCR was performed using SYBR Green master mix and run on a CFX96 Bio-Rad real time PCR machine. Primer sequences for 45S pre-rRNA are forward 5′ CCGCGCTCTACCTTACCTACCT 3′, reverse 5′ GCATGGCTTAATCTTTGAGACAAG 3′; for βActin are forward 5′ CGTCACCAACTGGGACGACA 3′, reverse 5′ CTTCTCGCGGTTGGCCTTGG 3′. Experiments were repeated three times. Results were normalized to GAPDH and β Actin expression for each sample.

## SUPPLEMENTARY FIGURE


