# Supplementary material for: Transient rRNA synthesis inhibition with CX-5461 is sufficient to elicit growth arrest and cell death in acute lymphoblastic leukemia cells
Source: Oncotarget. 2015 Oct 12;6(33):34846–58. doi: 10.18632/oncotarget.5413 (PMC4741494; doi:10.18632/oncotarget.5413)
Supplement: Supplementary file 1 [file oncotarget-06-34846-s001.pdf]

SUPPLEMENTARY FIGURE

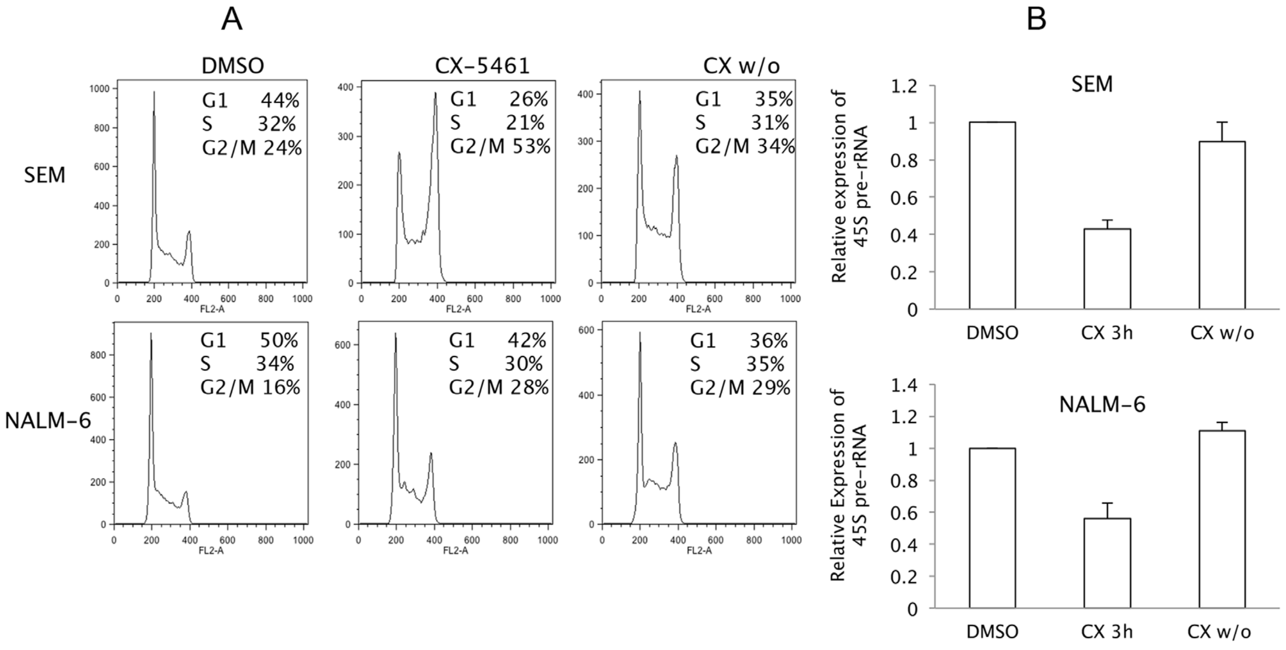

C

	CI		
	ED50	ED75	ED90
CX-5461:UCN-01	0.57769	0.68772	0.81912
CX-5461:Trametinib	0.51392	0.59595	0.69108

Supplementary Figure S1: Experiment was performed as in figure 2a. Middle panel shows 24 hours continuous CX-5461 drug treatment. Representative flow cytometry data is shown from one of the three experiments. **b.** 45S pre-rRNA transcript levels were measured using quantitative PCR and normalized to the expression of GAPDH and Actin. 3 hours CX-5461 treatment substantially reduced the levels of 45S transcript while drug washout cells (CX w/o) show complete recovery of rRNA synthesis at 24 hours. **c.** Combination indices for drug treated NALM-6 cells were calculated using the method of Chou and Talalay (1).

REFERENCE

1. Chou TC, Talalay P. Quantitative analysis of dose-effect relationships: the combined effects of multiple drugs or enzyme inhibitors. Adv Enzyme Regul. 1984; 22:27–55
